# Serum C-peptide level and the risk of cardiovascular diseases mortality and all-cause mortality: a meta-analysis and systematic review

**DOI:** 10.3389/fcvm.2023.1205481

**Published:** 2023-07-07

**Authors:** Hamid Ahmadirad, Farshad Teymoori, Ebrahim Mokhtari, Mitra Kazemi Jahromi, Mostafa Norouzzadeh, Saeed Tavakkoli, Tahere Shahrokhtabar, Hossein Farhadnejad, Parvin Mirmiran

**Affiliations:** ^1^ Nutrition and Endocrine Research Center, Research Institute for Endocrine Sciences, Shahid Beheshti University of Medical Sciences, Tehran, Iran; ^2^ Department of Nutrition, School of Public Health, Iran University of Medical Sciences, Tehran, Iran; ^3^ Department of Community Nutrition, School of Nutrition and Food Sciences, Shiraz University of Medical Sciences, Shiraz, Iran; ^4^ Endocrinology and Metabolism Research Center, Hormozgan University of Medical Sciences, BandarAbbas, Iran

**Keywords:** cardiovascular diseases mortality, all-cause mortality, mortality, C-peptide, meta-analysis

## Abstract

**Aims and background:**

Recently, the serum of C-peptide has been the focus of researchers as a possible predictor of mortality. However, the possible association of serum C-peptide with cardiovascular diseases (CVDs) mortality and all-cause mortality has not been clearly identified. This meta-analysis aimed to assess the relationship between serum C-peptide and the risk of CVDs mortality and all-cause mortality.

**Methods:**

A comprehensive and systematic search was performed in various important databases, including the PubMed, Web of Science, and Scopus to find relevant studies up to November 2022. The reported hazard ratio (HR) [95% confidence interval (CI)] for all studies was converted into log HR, and their SD was calculated. Then to compute the pooled HR, the random-effects model with inverse variance weighting method was performed.

**Results:**

Twenty-three studies were included in the meta-analysis. Fourteen studies reported HR for all-cause mortality, and nine studies for CVDs-related mortality. The pooled results indicate a significant association between serum C-peptide and the risk of all-cause mortality (HR: 1.22; 95% CI: 1.12–1.32, *I*^2 ^= 76.8%; *P*-value < 0.001). Also, higher serum C-peptide was related to the increased risk of CVDs mortality (HR: 1.38; 95% CI: 1.08–1.77, *I*^2 ^= 81.8%; *P*-value = 0.012).

**Conclusions:**

Our investigation suggested that an increased level of serum C-peptide is associated with a higher risk of both CVDs and all-cause mortality. Further, large-scale studies and sufficient samples are recommended to present a convincing link between C-peptide and the risk of CVDs and all-cause mortality.

**Systematic Review Registration:**

identifier, CRD42022364842.

## 1. Introduction

Cardiovascular diseases (CVDs), as a major health problem, are defined as any disease of the heart and its associated blood vessels such as angina, myocardial infarction, heart failure, and stroke (1). CVDs are considered the major cause of morbidity and mortality worldwide; it is estimated to be responsible for 31.8% of all deaths (2). During the recent decade, the number of deaths from CVDs has increased by 12.5% globally (2, 3). In addition to the genetic background, hypertension, diabetes, dyslipidemia, obesity, smoking, unhealthy diet, and sedentary lifestyle are considered as the main risk factors for the occurrence of CVDs events and CVDs-related mortality (4). Despite the advances in the prevention and treatment, the burden of CVDs remains high, and there is a need for better risk assessment tools to identify the individuals at high risk of CVDs.

Insulin resistance (IR) and hyperinsulinemia (HI) are two hallmarks and risk factors for CVDs events (5–8), by being involved in the pathogenesis of obesity and diabetes (9, 10). Studies have shown that serum insulin can predict the occurrence of CVDs (11, 12). C-peptide, a 31-amino acid peptide and one of the components of proinsulin, is secreted from the beta cells of the pancreas at an amount equimolar with insulin, and it is used to measure secreted insulin (13). Although C-peptide has been known to be biologically inactive, it is now considered as a marker of IR and a useful indicator of β-cell function. Also, recent studies have shown that high levels of C-peptide are associated with cardiometabolic disorders in individuals with IR and diabetes, such as diabetic vasculopathy and atherogenesis (14, 15). Many studies have investigated the relationship between C-peptide with all-cause (16–29) and CVDs-related mortality (17–19, 21, 23, 27, 29, 30), which indicated interesting results. Most of the conducted studies revealed that serum C-peptide levels have a significant direct relationship with all-cause (16–23) and CVDs-related mortality (17–19, 21, 23, 29); however, a study indicated an inverse association between C-peptide and CVDs mortality (30). Furthermore, some other studies showed no significant association between serum C-peptide levels with all-cause (24–29) and CVDs mortality (23, 27). Considering that the evidence on this association is inconsistent, a systematic review and meta-analysis are needed to provide a comprehensive assessment of the available evidence.

Due to the lack of a comprehensive meta-analysis summarizing all available findings in this area, as well as the existence of conflicting results in relevant studies, we conducted the present meta-analysis to summarize the available evidence on the association between serum C-peptide levels with all-cause and CVDs-related mortality. The findings of this meta-analysis will provide important insights into the potential role of serum C-peptide levels as a biomarker for predicting the risk of CVDs mortality and all-cause mortality. This information can be useful for developing risk assessment tools and for identifying the individuals who may benefit from targeted interventions to reduce their risk of CVDs mortality and all-cause mortality.

## 2. Materials and methods

### 2.1. Search strategy

This systematic review and meta-analysis used the Preferred Reporting Items for Systematic Reviews and Meta-Analyses to report the study findings (Supplementary Figure S3) (31). To find the published literature up to November 2022, we explored multiple electronic databases online including the PubMed, Scopus, and Web of Science without any language or study design limitation. We conducted a structured search using the following related MeSH terms and keywords: “C-Peptide” or “proinsulin” combined with “cardiovascular” or “heart disease” or “mortality” or “death” or “survival” or “overall mortality” or “coronary artery” (Supplementary Table S2). The reference lists of all obtained studies were hand-searched to avoid missing any publications. This meta-analysis is registered on the International Prospective Register of Systematic Reviews (PROSPERO) under the registration number CRD42022364842.

### 2.2. Inclusion and exclusion criteria

Initially, we scanned the title and abstract of all studies; then those studies that have examined the association between serum C-peptide levels with all-cause and CVDs-related mortality underwent full-text screening. Studies were included in the current meta-analysis if they were: (1) the original article, (2) cohort design, and (3) reported hazard ratio (HR) with 95% confidence interval (CI). Randomized clinical trials, reviews, meta-analyses, cross-sectional studies, unpublished data, and gray literature including congress abstracts, patents, and dissertations were excluded. PICO (patients, intervention/exposure, comparison, outcome) criteria are presented in Supplementary Table S1.

### 2.3. Data extraction

To collect the required information from each study and increase the accuracy, two reviewers conducted the data extraction. The following information was extracted from all included studies: name of the author, the publication date of the study, location, target population, sample size, number of cases, age range and sex of the participants, the method used for C-peptide assessment, outcome, compared categories, HR with 95% CI for C-peptide, adjusted covariates, and the follow-up duration.

### 2.4. Validity and quality assessment of studies

A methodological quality assessment of the included studies was assessed using the Newcastle–Ottawa scales (*N*OS) designed for the cohort design by two reviewers, independently (32). The NOS has 9 points and is categorized into 3 groups: low (less than 4 scores), moderate (between 4 and 6 scores), and high quality (higher than 6 scores) (Supplementary Table S3).

### 2.5. Statistical analyses

We used HR with 95% CI to analyze dichotomous data (all-cause mortality and CVDs-related mortality). The present meta-analysis includes 15 eligible papers that consist of 23 cohorts. A total of 14 of these cohorts investigated the association between C-peptide levels with the risk of all-cause mortality; however, other studies examined the association with CVDs-related mortality. All included studies reported the measure of association as HR (95% CI). Seven of the 23 studies reported the HR of CVDs and all-cause mortality between the highest and lowest categories of C-peptide level; however, others compared the risk of both outcomes for one unit or one standard deviation (SD) increasing the C-peptide levels. The reported HR (95% CI) for all studies was converted into log HR, and their SD was calculated. Then to compute the pooled HR, the random-effects model with inverse variance weighting method was performed.

The *I*^2^ quantity (which values greater than 50% represented a significant heterogeneity) (33) and Cochrane's *Q* statistics (*P*-value < 0.10 considered as significant) (34) were used for examining the heterogeneity between studies and the statistical significance levels of heterogeneity, respectively. In our meta-analyses, the test for heterogeneity was statistically significant. Thus, to find the potential sources of heterogeneity, subgroup analyses were conducted based on region, cancer, diabetes status, and type of exposure analysis for all-cause mortality, and region, diabetes, and type of exposure analysis for CVDs mortality using the random-effects method. Due to the number of included studies being more than 10, we carried out random-effects univariate and multivariate meta-regression to examine the role of potential sources of heterogeneity related to the participants (age, year, region, follow-up period, sample size, cancer, and diabetes status) for both outcomes. The publication bias was examined by the visual inception of the funnel plot, Egger's regression test, and Begg's adjusted rank correlation test. Also, to simulate the required studies, the trim-and-fill method was used. A sensitivity analysis was performed by removing each study and recalculating the pooled effect size (i.e., one-study-removed analysis). All statistical analyses were carried out using the Stata version 11.2 software, and *P* < 0.05 was considered statistically significant. All statistical tests were two-sided.

## 3. Results

### 3.1. Literature search

**Figure 1:**
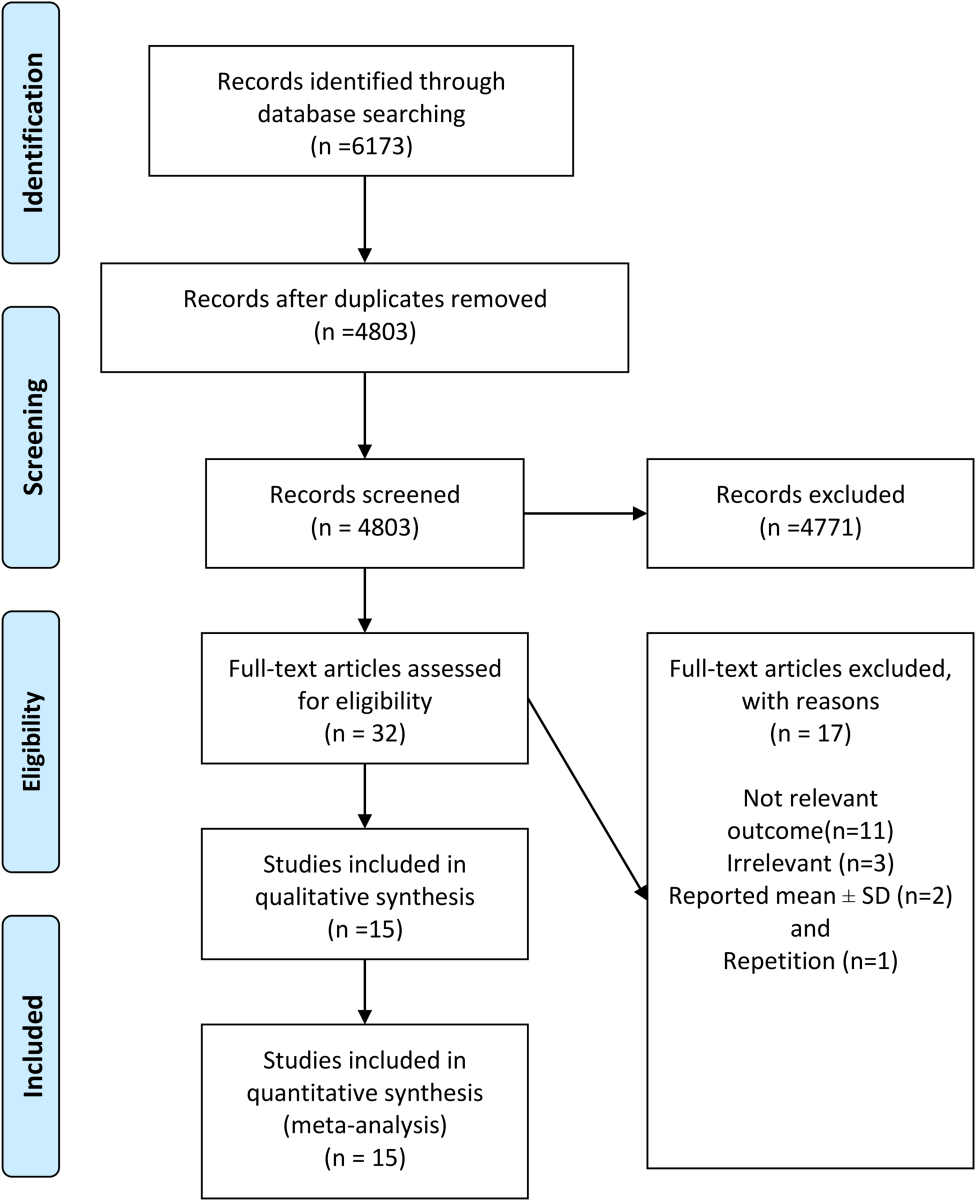
Flow diagram of selection of the published studies.

As presented in Figure 1, a total of 6,173 publications were initially identified. After excluding duplicate and irrelevant articles, 32 full-text papers of potentially relevant studies were identified. After full-text review, papers were excluded due to the following reasons: not relevant outcome (*n* = 11), irrelevant (*n* = 3), reported mean ± SD (*n* = 2), and repetition (*n* = 1). Finally, 15 papers including 23 studies were included in the meta-analysis. Fourteen studies reported HR for all-cause mortality and nine studies for CVDs-related mortality.

### 3.2. Characteristics of included studies

The characteristics of the 23 included studies are reported in Table 1. The studies were published between 2003 and 2020 and were conducted in the United States (*n* = 12), Italy (*n* = 5), Germany (*n* = 2), Sweden (*n* = 2), New Zealand (*n* = 1), and China (*n* = 1). The number of participants in the studies investigated the association between serum C-peptide, with the all-cause and CVDs-related mortality ranging from 84 to 5,902 and with an age range of 18–91 years. During the follow-up period that ranged from 1.46 to 17 years, a total of 9,530 deaths occurred (all-cause deaths: *n* = 6,983; CVDs-related deaths: *n* = 2,547).

**Table 1:** Characteristics of included cohort studies in the meta-analysis.

Studies	Country	Target population	Sample size/cases	Gender, age range	Exposure assessment	Outcome	Comparison	HR (95% CI)	Adjustment for covariate	Follow-up (years)	NOS scores
Marx et al. (21)	Germany	Undergoing angiography	2,306/440	M, F 61.5 ± 11.2 years	Enzyme immunoassay	All-cause mortality	C-peptide (T3 vs. T1)	HR = 1.46 (1.10–1.93)	Age, sex, BMI, hypertension, smoking, GFR, triglycerides, LDL cholesterol, and HDL cholesterol, fasting glucose, fasting insulin, glycated hemoglobin, newly diagnosed type 2 diabetes per American Diabetes Association 2,010 guidelines, proinsulin, free fatty acids, free glycerol, and CRP.	7.6 years	9/9
Marx et al. (21)	Germany	Undergoing angiography	2,306/252	M, F 61.5 ± 11.2 years	Enzyme immunoassay	CVD mortality	C-peptide (T3 vs. T1)	HR = 1.55 (1.07–2.24)	Age, sex, BMI, hypertension, smoking, GFR, triglycerides, LDL cholesterol, and HDL cholesterol, fasting glucose, fasting insulin, glycated hemoglobin, newly diagnosed type 2 diabetes per American Diabetes Association 2,010 guidelines, proinsulin, free fatty acids, free glycerol, and CRP.	7.6 years	9/9
Pikkemaat et al. (17)	Sweden	Diabetic	398/104	M, F 52.4 ± 8.7 years	Laboratory analyses	All-cause mortality	C-peptide per 1 nmol/L	HR = 2.20 (1.49–3.25)	Age, sex, smoking, SBP, HbA1c, antihypertensive treatment, BMI, CRP, eGFR, cholesterol, and previous myocardial infarction or ischemic stroke.	17 years	8/9
Pikkemaat et al. (17)	Sweden	Diabetic	398/35	M, F 52.4 ± 8.7 years	Laboratory analyses	CVD mortality	C-peptide per 1 nmol/L	HR = 2.69 (1.49–4.85)	Age, sex, smoking, SBP, HbA1c, antihypertensive treatment, BMI, CRP, eGFR, cholesterol, and previous myocardial infarction or ischemic stroke.	17 years	8/9
Hirai et al. (23)	USA	Diabetic	1,007/824	M, F 68.6 ± 11.0 years	N/A	All-cause mortality	C-peptide per 1 nmol/L	HR = 1.15 (1.04–1.29)	Age, sex, BMI, diabetes duration, SBP, history of cardiovascular disease, presence of diabetic retinopathy, cigarette smoking, time since last meal, exogenous insulin use, and glycosylated hemoglobin.	16 years	8/9
Hirai et al. (23)	USA	Diabetic	1,007/358	M, F 68.6 ± 11.0 years	N/A	Ischemic heart mortality	C-peptide per 1 nmol/L	HR = 1.19 (1.02–1.39)	Age, sex, BMI, diabetes duration, SBP, history of cardiovascular disease, presence of diabetic retinopathy, cigarette smoking, time since last meal, exogenous insulin use, and glycosylated hemoglobin.	16 years	8/9
Hirai et al. (23)	USA	Diabetic	1,007/137	M, F 68.6 ± 11.0 years	N/A	Stroke mortality	C-peptide per 1 nmol/L	HR = 1.09 (0.85–1.40)	Age, sex, BMI, diabetes duration, SBP, history of cardiovascular disease, presence of diabetic retinopathy, cigarette smoking, time since last meal, exogenous insulin use, and glycosylated hemoglobin.	16 years	8/9
Patel et al. (18)	USA	General	5,152/932	M, F 40–74 years	N/A	All-cause mortality	C-peptide per 1 nmol/L	HR = 1.72 (1.34–2.21)	Age, sex, race, waist-to-hip ratio, BMI, blood pressure (normal, prehypertension, hypertension), total cholesterol, triglycerides, HDL, presence or absence of past history of stroke, heart attack, peripheral arterial disease, presence or absence of family history of diabetes and heart attack, history of chest pain (suggestive of angina, not suggestive of angina and no chest pain), level of education, smoking status, level of physical activity, alcohol use, CRP level, urinary albumin/creatinine ratio, GFR, and glycated hemoglobin levels.	14.4 years	8/9
Patel et al. (18)	USA	General	5,140/370	M, F 40–74 years	N/A	CVD mortality	C-peptide per 1 nmol/L	HR = 1.60 (1.07–2.39)	Age, sex, race, waist-to-hip ratio, BMI, blood pressure (normal, prehypertension, hypertension), total cholesterol, triglycerides, HDL, presence or absence of past history of stroke, heart attack, peripheral arterial disease, presence or absence of family history of diabetes and heart attack, history of chest pain, level of education, smoking status, level of physical activity, alcohol use, CRP level, urinary albumin/creatinine ratio, GFR, and glycated hemoglobin levels.	14.4 years	8/9
Min et al. (19)	USA	General	5,902/2,020	M, F 40- 70 years	Radioimmunoassay	All-cause mortality	C-peptide per 1 unit	HR = 1.80 (1.33–2.43)	Age, sex, ethnic background, education, smoking status, alcohol consumption status, day on which the last meal or snack was consumed, history of hypertension, hypercholesterolemia, heart attack and BMI, CRP, total cholesterol, triglyceride, serum insulin, glycated hemoglobin and fasting serum glucose levels.	–	8/9
Min et al. (19)	USA	General	5,902/895	M, F 40- 70 years	Radioimmunoassay	CVD mortality	C-peptide per 1 unit	HR = 3.20 (2.07–4.93)	Age, sex, ethnic background, education, smoking status, alcohol consumption status, day on which the last meal or snack was consumed, history of hypertension, hypercholesterolemia, heart attack and BMI, CRP, total cholesterol, triglyceride, serum insulin, glycated hemoglobin and fasting serum glucose levels.	–	8/9
Cardellini et al. (29)	Italy	Atherosclerotic	268/44	M, F 31–91 years	Immunochemiluminescence assay	All-cause mortality	C-peptide per SD	HR (97/5%CI) = 1.09 (0.90–1.32)	Age, sex, diabetes treatment, GFR and known diabetes status.	4.6 years	8/9
Cardellini et al. (29)	Italy	Atherosclerotic	268/20	M, F 31–91 years	Immunochemiluminescence assay	CVD mortality	C-peptide per SD	HR (97/5%CI) = 1.38 (1.05–1.80)	Age, sex, diabetes treatment, GFR and known diabetes status.	4.6 years	8/9
Volkova et al. (28)	New Zealand	Cancer	344/91	M, F 31–91 years	Immunoassay	Survival (mortality)	C-peptide	HR = 1.02 (0.96–1.07)	-	10 years	8/9
Wolpin et al. (22)	USA	Cancer	350/98	M, F 30–75 years	Enzyme-linked immunosorbent assay	Overall mortality	C-peptide (Q4 vs. Q1)	HR = 2.11 (1.06–4.21)	Age at diagnosis, cohort (sex), stage of disease, histologic differentiation, tumor location, time period of diagnosis, time between last meal and plasma collection, receipt of chemotherapy, and patient characteristics from the most recent questionnaire before blood draw, including smoking status, aspirin use, alcohol consumption (g/d), total vitamin D intake (U/d), and postmenopausal hormone use.	5.9 years	9/9
Rovere et al. (30)	Italy	Diabetic	113/22	M, F 66.9 ± 9.9 years	Specific immunoassay	Macrovascular mortality	C-peptide	HR = 0.25 (0.08–0.74)	-	9 years	9/9
Schrauben et al. (25)	USA	General	1,883/379	M, F 56.5 years	NA	All-cause mortality	C-peptide per 1 SD	HR = 1.09 (0.94–1.27)	Age, sex, race, ethnicity, level of education, clinical center, BMI, waist circumference, smoking status, SBP, ACEi/ARB use, HDL, LDL, triglycerides, high sensitivity CRP, fat-free mass, eGFR, hemoglobin, physical activity, use of statins, use of other lipid-lowering medications, history of CVD, 24-h urine protein, FGF-23, uric acid, serum albumin.	7.7 years	9/9
Guercio et al. (26)	USA	Cancer	1,086/932	M, F 59 yeras	Enzyme-linked immunosorbent assays	Overall survival	C-peptide (Q5 vs. Q1)	HR = 1.13 (0.91- 1.40)	Age, sex, performance status, planned chemotherapy, prior adjuvant chemotherapy, assigned treatment arm, KRAS status, tumor sidedness, plasma albumin, diabetes, BMI, and fasting status.	6.2 years	8/9
Bo et al. (27)	Italy	Diabetic	2,112/973	M, F 66 years	Enzyme immunoassay	All-cause mortality	C-peptide (T3 vs. T1)	HR = 1.10 (0.93–1.30)	Age, sex, BMI, smoking, time since diagnosis, insulin treatment, values of HbA1c, SBP, HDL-cholesterol and triglycerides, presence of retinopathy, nephropathy, neuropathy and CVDs.	14 years	9/9
Bo et al. (27)	Italy	Diabetic	2,113/458	M, F 66 years	Enzyme immunoassay	CVD mortality	C-peptide (T3 vs. T1)	HR = 0.92 (0.73–1.17)	Age, sex, BMI, smoking, time since diagnosis, insulin treatment, values of HbA1c, SBP, HDL-cholesterol and triglycerides, presence of retinopathy, nephropathy, neuropathy and CVDs.	14 years	9/9
Zhu et al. (24)	China	Cancer	2,682/55	F, 55.3 ± 12.4 years	Electrochemiluminescence immunoassay	All-cause mortality	C-peptide high vs. low	HR = 1.14 (0.63–2.08)	Age, BMI, menopausal status, tumor size, lymph node status, chemotherapy, radiotherapy, endocrine therapy, and targeted therapy.	3.1 years	8/9
Irwin et al. (20)	USA	Cancer	604/64	F, >18 years	Radioimmunoassay method	All-cause mortality	C-peptide (ng/ml)	HR = 1.31 (1.06–1.63)	Age, race/site, and initial treatment; BMI, disease stage, and estrogen receptor status, and women with type 2 diabetes.	6 years	8/9
Griffith et al. (26)	USA	Diabetic	84/27	M-F > 18 years	–	Overall survival	C-peptide (ng/ml)	HR = 1.05 (1.01–1.09)	Peak steroid dose, myeloablative conditioning.	1.46 years	8/9

BMI, body mass index; CI, confidence interval; CRP, C-reactive protein; CVDs, cardiovascular diseases; FGF-23, fibroblast growth factor 23; F, female; GFR, glomerular filtration rate; HDL, high-density lipoprotein; HR, hazard ratio; HbA1c, hemoglobin A1C; LDL, low-density lipoprotein; NOS, Newcastle–Ottawa scale; M, male; Q, quartile; SD, standard deviation; SBP, systolic blood pressure; T, tertile.

The studies included females (*n* = 2) and both genders (*n* = 21). As previously mentioned, all cohort studies reported the measure of association as HR and 95% CI.

Most studies controlled for some conventional risk factors, including age (*n* = 20), sex (*n* = 18), underlying disease (*n* = 17), smoking (*n* = 15), BMI (*n* = 15), blood pressure (*n* = 10), glomerular filtration rate (*n* = 9), triglycerides (*n* = 7), and alcohol consumption (*n* = 5). All studies had high quality based on the NOS criteria (Supplementary Table S3).

### 3.3. Meta-analysis

#### 3.3.1. C-peptide and all-cause mortality

**Figure 2:**
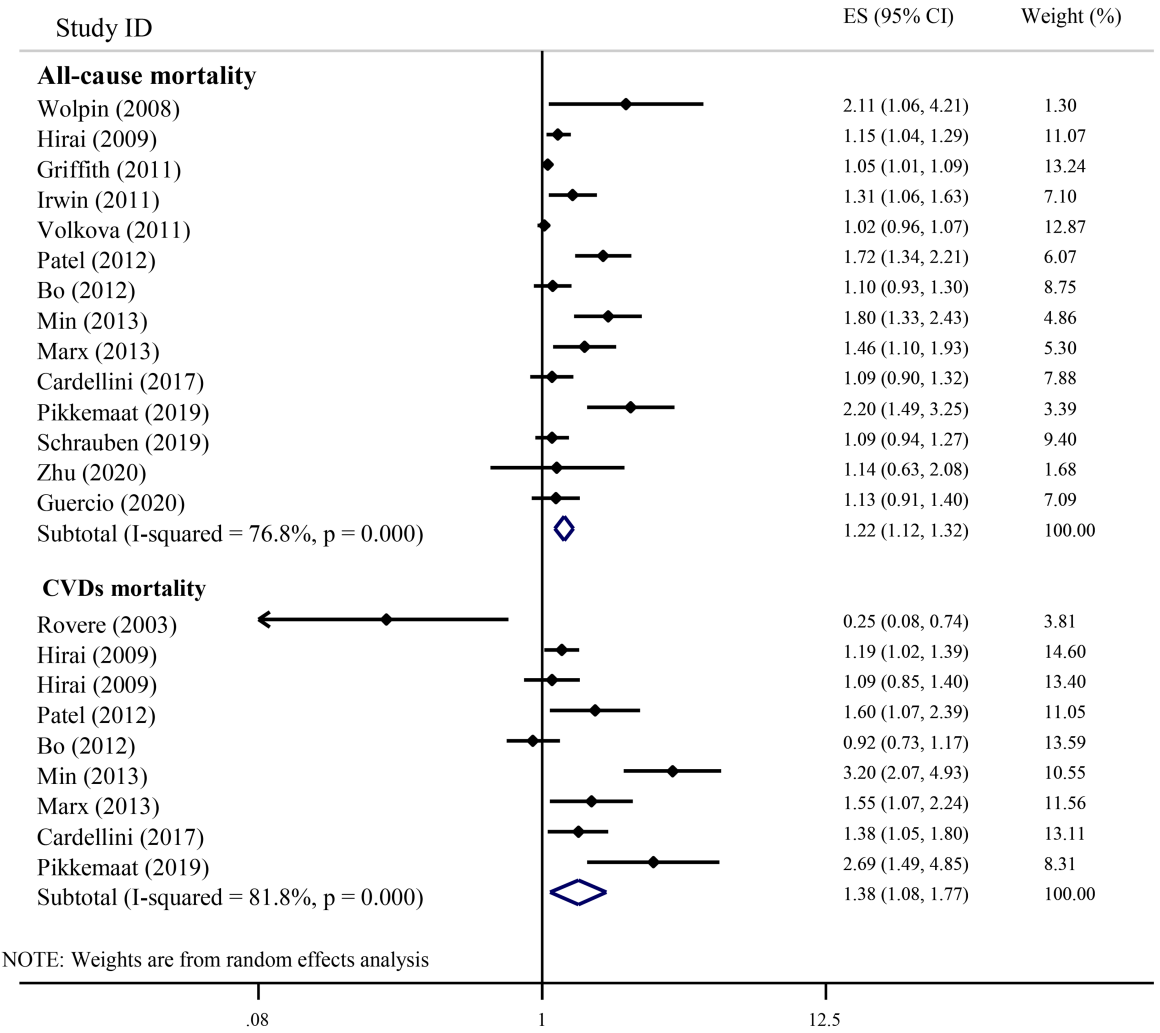
The association of serum C-peptide levels with the risk of all-cause and CVDs mortality in cohort studies.

Figure 2 shows the results of the individual studies and the overall summary estimate of HR (95% CI) for the relationship between serum C-peptide and all-cause mortality. The range of HR across the studies was 1.02–2.20. Eight studies reported an increased all-cause mortality in individuals with higher serum C-peptides, but six studies did not observe significant findings. The pooled (overall) HR (95% CI) using the random-effects model was 1.22 (1.12, 1.32). There was a significant heterogeneity among studies (*I*^2^ = 76.8%; *P*-heterogeneity < 0.001).

A subgroup analysis was performed based on the region (studies conducted in the United States: *n* = 8, Europe: *n* = 4, Oceania: *n* = 1, and Asia: *n* = 1), diabetes status (studies conducted on diabetic: *n* = 4, and non-diabetic patients: *n* = 10), cancer status (cancer: *n* = 5 vs. non-cancer patients: *n* = 9), and type of exposure analysis (per 1 unit: *n* = 9, highest vs. lowest category; *n* = 5) to find the source of heterogeneity in the studies that assessed the association between serum C-peptide and the risk of all-cause mortality (Table 2). We observed a significant heterogeneity between subgroups of the region of study; therefore, the study region is known as the source of heterogeneity. The pooled HR (95% CI) for serum C-peptide with all-cause mortality was 1.25 (1.11–1.42) and 1.33 (1.04–1.71) in the studies conducted in the United States and Europe, respectively. However, there was no significant association between serum C-peptide with all-cause mortality in the studies conducted in Asia and Oceania.

**Table 2:** Summary hazard ratio (HR) estimates [95% confidence intervals (CIs)] for sub-group analysis of the association between the serum C-peptide levels with the all-cause and CVDs mortality.

Subgroups	Study numbers	Summary HR (95% CI)	Between studies		Between subgroups	
			*I* ^2^	*P*_heterogeneity_	*Q*	*P*_heterogeneity_
All-cause mortality						
Region					8.54	0.036^a^
United States	8	1.25 (1.11–1.42)	79.7	0.000		
Europe	4	1.33 (1.04–1.71)	77.2	0.004		
Oceania	1	1.02 (0.96–1.07)	0.0	0.0		
Asia	1	1.14 (0.62–2.07)	0.0	0.0		
Diabetes status					0.49	0.485^b^
Diabetic	4	1.17 (1.01–1.35)	81.1	0.001		
Non-diabetic	10	1.27 (1.11–1.46)	77.4	0.000		
Cancer status					1.89	0.170^c^
Cancer	5	1.16 (0.98–1.36)	58.2	0.048		
Non-cancer	9	1.27 (1.12–1.43)	82.1	0.000		
Type of exposure analysis					2.84	0.092
Per 1 unit	9	1.22 (1.10–1.34)	83.2	0.000		
Highest vs. lowest category	5	1.22 (1.04–1.42)	30.7	0.217		
CVDs mortality						
Region					0.52	0.469^d^
United States	4	1.53 (1.06–2.22)	85.5	0.000		
Europe	5	1.22 (0.80–1.86)	82.3	0.000		
Diabetes status					14.33	0.000^b^
Diabetic	5	1.11 (0.82–1.50)	78.7	0.001		
Non-diabetic	4	1.77 (1.26–2.50)	72.1	0.013		
Type of exposure analysis					3.25	0.072
Per 1 unit	7	1.46 (1.07–1.98)	82.9	0.000		
Highest vs. lowest category	2	1.17 (0.70–1.95)	81.6	0.020		

All statistical tests were two-sided.

^a^ Test for heterogeneity between studies conducted on the different regions including the United States, Europe, Oceania, and Asia.

^b^ Test for heterogeneity between studies conducted on diabetes status (diabetic or non-diabetic).

^c^ Test for heterogeneity between studies conducted on cancer status (cancer or non-cancer).

^d^ Test for heterogeneity between studies conducted on the different regions including the United States and Europe.

#### 3.3.2. C-peptide and CVDs mortality

Figure 2 indicates the results of the individual studies and the overall summary estimate of HR (95% CI) for the relationship between serum C-peptide and CVDs-related mortality. Of the nine included studies, six studies reported a significant elevation in CVDs-related mortality with an elevation of serum C-peptide. However, two studies showed non-significant results, and just one study reported a risk reduction with an elevation of serum C-peptide. The range of individual HRs between studies was 0.25–3.20, and the summary HR (95% CI) for all studies combined was 1.38 (1.08, 1.77). The heterogeneity among studies was statistically significant (*I*^2^ = 81.8%; *P*-heterogeneity < 0.001).

A subgroup analysis was performed based on the region (studies conducted in the United States: *n* = 4, and Europe: *n* = 5), diabetes status (studies on diabetic: *n* = 5, and non-diabetic patients: *n* = 4), and type of exposure analysis (per 1 unit: *n* = 7, highest vs. lowest category; *n* = 2) to find the source of heterogeneity of studies that assessed the association between serum C-peptide and risk of CVDs-related mortality. According to the results given in Table 2, a significant heterogeneity was found between subgroups of the diabetic status of the study population; therefore, the diabetic status is known as the source of heterogeneity. The pooled HR (95% CI) for serum C-peptide with CVDs-related mortality was 1.11 (0.82–1.50) and 1.77 (1.26–2.50) for diabetic and non-diabetic patients, respectively. However, there was no significant association between serum C-peptide with CVDs-related mortality in the studies that worked on diabetic patients.

#### 3.3.3. Meta-regression for all-cause mortality

We fit a meta-regression model including the age of participants and other covariates of interest. The model showed that a great percentage of this heterogeneity can be explained by the sample size of the study (Table 3). Likewise, a significant positive association was observed for the sample size of the study in the unadjusted model (coefficient: 1.00; 95% CI: 1.00, 1.00; *P *= 0.014). This finding suggests that for each participant increase in the sample size, the pooled HR increases by 1.00008-fold. Even by adjusting the age, year, cancer status, and region, the significant reduction effect of the sample size on the *τ*^2^ remained. Although after adding more than one covariate to the adjusted model, the sample size effect was attenuated and become non-significant, it may be influenced by the low number of included studies as it needed to add some covariates for adjustment.

**Table 3:** Meta-regression model with the hazard ratio (95% CI) of the all-cause mortality as a dependent variable^a^.

Variable	Unadjusted meta-regression model			Adjusted meta-regression model			*τ* ^2^
	Coefficient	95% CI	*P*	Coefficient	95% CI	*P*	
Without any covariate							0.034
Univariate model							
Age	0.99	(0.97–1.00)	0.34				0.364
Year	0.99	(0.96–1.03)	0.98				0.040
Region							
United States	1.04	(0.77–1.40)	0.75				0.039
Europe	1.06	(0.77–1.47)	0.65				0.038
Follow-up	1.00	(0.97–1.03)	0.58				0.038
Sample size	1.00	(1.00–1.00)	0.014				0.004
Diabetes status	0.95	(0.69–1.29)	0.72				0.039
Cancer status	0.92	(0.67–1.25)	0.57				0.037
**Model 1**							0.012
Sample size				1.00	(1.00–1.00)	0.04	
Age				0.99	(0.98–1.00)	0.56	
**Model 2**							
Sample size				1.00	(0.99–1.00)	0.07	
Age				0.99	(0.97–1.01)	0.56	
Year				0.99	(0.96–1.03)	0.92	
**Model 3**							0.025
Sample size				1.00	(0.99–1.00)	0.10	
Age				0.99	(0.97–1.01)	0.49	
Year				0.99	(0.95–1.03)	0.83	
United States				0.93	(0.66–1.32)	0.69	
**Model 4**							0.023
Sample size				1.00	(0.99–1.00)	0.09	
Age				0.99	(0.97–1.01)	0.40	
Year				0.99	(0.95–1.03)	0.73	
Europe				1.13	(0.82–1.57)	0.38	
**Model 5**							0.035
Sample size				1.00	(0.99–1.00)	0.20	
Age				1.00	(0.96–1.01)	0.51	
Year				0.99	(0.94–1.04)	0.78	
United States				0.93	(0.63–1.37)	0.69	
Diabetes status				0.97	(0.65–1.45)	0.88	
**Model 6**							0.029
Sample size				1.00	(0.99–1.00)	0.20	
Age				0.99	(0.96–1.01)	0.35	
Year				0.98	(0.94–1.03)	0.57	
Europe				1.18	(0.80–1.75)	0.33	
Diabetes status				0.91	(0.60–1.36)	0.61	
**Model 7**							0.034
Sample size				1.00	(0.99–1.00)	0.16	
Age				0.99	(0.96–1.01)	0.50	
Year				0.99	(0.95–1.04)	0.80	
United States				0.93	(0.63–1.37)	0.69	
Cancer status				1.02	(0.70–1.47)	0.89	
**Model 8**							0.028
Sample size				1.00	(0.99–1.00)	0.09	
Age				0.98	(0.96–1.01)	0.30	
Year				0.98	(0.94–1.03)	0.58	
Europe				1.24	(0.81–1.88)	0.26	
Cancer status				1.14	(0.76–1.71)	0.46	

^a^ The meta-regression model is based on the ReML estimator method. Initial heterogeneity without any variable assigned in the model was *τ*^2^ = 0.034. “Age” indicates the mean age; “Duration” indicates the duration of follow-up in years; “Sample size” indicates the total sample size; “Year” indicates the year of study publication; “Coefficient” indicates the regression coefficient; and “*τ*^2^” indicates the between-study variance in a random-effects meta-analysis.

#### 3.3.4. Meta-regression for CVD-related mortality

We fit a meta-regression model including the age of participants and other covariates of interest. The model showed that a great percentage of this heterogeneity can be explained by the year of publication (Table 4) (Supplementary Figure S2). In this context, a significant positive association was observed for the year of publication in the unadjusted model (coefficient: 1.10; 95% CI: 1.00, 1.20; *P *= 0.04). This finding suggests that for each unit increase in the year of publication, the pooled HR increases by 1.10-fold. However, adjusting the possible confounding variables lead to a disappearance of the significant association observed for the year of publication. In all adjusted models for different variables, the *τ*^2^ was significantly decreased.

**Table 4:** Meta-regression model with the hazard ratio (95% confidence interval) of cardiovascular diseases mortality as a dependent variable^a^.

Variable	Unadjusted meta-regression model			Adjusted meta-regression model			*τ* ^2^
	Coefficient	95% CI	*P*	Coefficient	95% CI	*P*	
Without any covariate							0.213
Univariate model							
Age	0.95	(0.90–1.00)	0.05				0.069
Year	1.10	(1.00–1.20)	0.04				0.131
Region							
United States vs. other	1.31	(0.48–3.55)	0.54				0.265
Europe vs. other	0.76	(0.28–2.07)	0.54				0.265
Follow-up	0.97	(0.89–1.06)	0.48				0.208
Sample size	1.00	(0.99–1.00)	0.18				0.157
Diabetes status	0.60	(0.25–1.44)	0.21				0.166
**Model 1**							0.063
Year				1.06	(0.97–1.16)	0.12	
Age				0.96	(0.91–1.00)	0.09	
**Model 2**							0.072
Year				1.06	(0.95–1.17)	0.20	
Age				0.96	(0.91–1.01)	0.12	
Follow-up				0.98	(0.92–1.04)	0.59	
**Model 3**							0.107
Year				1.09	(0.95–1.25)	0.14	
Age				0.98	(0.89–1.08)	0.71	
Follow-up				1.00	(0.92–1.08)	0.96	
Sample size				1.00	(0.99–1.00)	0.44	
**Model 4**							0.156
Year				1.11	(0.91–1.34)	0.17	
Age				0.99	(0.87–1.12)	0.84	
Follow-up				0.99	(0.87–1.13)	0.91	
Sample size				1.00	(0.99–1.00)	0.41	
Diabetes status				1.12	(0.19–7.75)	0.74	
**Model 5**							0.013
Year				1.10	(0.88–1.37)	0.20	
Age				0.96	(0.85–1.37)	0.37	
Follow-up				0.96	(0.84–1.10)	0.42	
Sample size				0.99	(0.99–1.00)	0.33	
Diabetes status				1.19	(0.25–5.69)	0.67	
United States vs. other				1.80	(0.61–5.34)	0.14	
**Model 6**							0.013
Year				1.10	(0.88–1.37)	0.20	
Age				0.96	(0.85–1.09)	0.50	
Follow-up				0.96	(0.84–1.10)	0.42	
Sample size				0.99	(0.99–1.00)	0.84	
Diabetes status				1.19	(0.25–5.69)	0.67	
Europe vs. other				0.55	(0.18–1.63)	0.14	

^a^ The meta-regression model is based on the ReML estimator method. Initial heterogeneity without any variable assigned in the model was *τ*^2^ = 0.213. “Age” indicates the mean age; “Duration” indicates the duration of follow-up in years; “Sample size” indicates the total sample size; “Year” indicates the year of the study publication; “Coefficient” indicates the regression coefficient; and “*τ*^2^” indicates the between-study variance in a random-effects meta-analysis.

### 3.4. Publication bias

A visual inspection of the funnel plot, Begg's test, and Egger's test indicated a publication bias in the association between serum C-peptide and the all-cause mortality (Supplementary Figure S1); however, there was no publication bias for the relation between serum C-peptide with CVDs-related mortality (*P*-value = 0.460) (Supplementary Figure S1). This should be interpreted with caution because our limitations about the number of studies were included in the analyses. Since there was a significant publication bias for the relation between serum C-peptide with all-cause mortality (*P*-value = 0.001), the trim-and-fill method was performed to calibrate publication bias. Two missing studies were identified by the trim-and-fill method for all-cause mortality. However, a minimal difference (0.04) was observed between the pooled estimate and random-effects model trimming estimation HR and 95% CI of model trimming estimation, which was 1.18 (1.08–1.29). Since this minimal difference does not affect the observed relationship, therefore, this amount of publication bias does not cause a problem.

### 3.5. Sensitivity analysis

According to the sensitivity analysis using a random-effects model, excluding none of the studies had a considerable change on the pooled effect size of the association between serum C-peptide and the all-cause mortality (range: 1.17–1.27) (Supplementary Table S4), and between serum C-peptide and CVDs-related mortality (range = 1.25–1.47) (Supplementary Table S5).

## 4. Discussion

### 4.1. Important findings

To the best of our knowledge, the present study is the first meta-analysis that compiled and reviewed the available literature from relevant observational studies that have assessed whether higher serum C-peptide level is associated with an increased risk of both CVDs and all-cause mortality. The present meta-analysis showed that higher serum C-peptide level was associated with an increased risk of all-cause mortality and CVDs mortality by 22% and 38%, respectively. Out of the 14 studies that have investigated all-cause mortality, eight studies have reported a positive relationship between serum C-peptide levels and all-cause mortality (16–23). Out of the nine studies that have investigated CVDs mortality, six studies have reported a positive relationship between serum C-peptide levels and CVDs mortality (17, 19, 21, 23, 29).

### 4.2. Literature review

Accumulating evidence from observational studies suggested that higher C-peptide serum level might increase all-cause mortality rates by increasing the risk of important chronic diseases, such as several types of cancer (35–38), CVDs, and major metabolic disorders (39, 40) that are the leading causes of death worldwide. In agreement with the findings of our meta-analysis, a recent systematic review suggested that higher serum levels of C-peptide were associated with an increased risk of breast cancer (35). Also, a study conducted on young adult in the framework of the Southern Brazilian cohort revealed that higher serum C-peptide level may be related to an increased risk of cardiometabolic disorders, including central adiposity, general obesity, hyperglycemia, and dyslipidemia (41). Furthermore, it is previously indicated that an unhealthy lifestyle, characterized by elevated level of body mass index, low physical activity, and poor dietary pattern, was associated with higher serum C-peptide levels, which can increase the risk of various diseases, such as several types of cancer (36–38), type 2 diabetes (39), and other metabolic disorders (39, 40); therefore, an unhealthy lifestyle may increase the risk of premature death and mortality rate by the mediation and interference in the possible relationship of C-peptide with the risk of chronic diseases in the long run. Moreover, another study showed that a dietary pattern associated with higher serum C-peptide levels was associated with poorer survival in patients with colorectal cancer (42). These figures from the previous studies support our finding that demonstrated increased level of serum C-peptide is linked to higher overall risk of death. It is worth mentioning that in the current meta-analysis, all the significant findings have demonstrated that higher serum C-peptide levels increase the risk of all-cause mortality, and the rest of the studies that reported a risk reduction were not significant. Also, regarding CVD mortality, except for one study (30), the rest have reported significant results of an increased risk. However, given the high heterogeneity, these findings should be declared with caution.

In the current study, the heterogeneity for all-cause mortality was 76% and for CVD mortality was 80%, and these results are significant. To further identify the source of heterogeneity, we performed subgroup analysis and meta-regression analysis. Based on the meta-regression analysis, the sample size in all-cause mortality and publication year in CVD mortality can be a positive source of heterogeneity. It is probable that in higher sample volumes and also with the updating of the measurement tool, the measurement of C-peptide level has been performed better, which helps people to be better classified, that is, people with different C-peptide levels can be better distinguished from each other, which makes the risk better detectable.

In the subgroup analysis for all-cause, we divided the studies into three subgroups, according to the location of residence, diabetes status, and cancer status. In CVD mortality, the studies were divided into two subgroups, according to the place of residence and diabetes status. Accordingly, in all-cause mortality, the location of residence is a source of heterogeneity, which is the reason for a large number of studies conducted in the United States and Europe compared with Asia and the Pacific. In CVDs mortality, the diabetes status is a source of heterogeneity. The reason for this could be that it is difficult to differentiate between people with different levels of C-peptide since the basic levels of C-peptide are high in all diabetics. However, in non-diabetic people, the difference between serum C-peptide levels is much clearer than that in diabetic people, and it is easier and better to separate people based on this. In such people, high levels of serum C-peptide indicate the presence of a metabolic disorder that can significantly increase the risk of mortality.

### 4.3. Biological plausibility

Several mechanisms have been proposed to explain the relationship between higher serum C-peptide levels and increased mortality. Insulin and C-peptide are secreted in equal proportion from pancreatic beta cells. C-peptide is a biomarker of insulin synthesis and was used as a surrogate biomarker of insulin in epidemiologic studies because it has a longer half-life than insulin and so is more stable (43). The current evidence shows the proatherogenic effects of C-peptide, which suggests that C-peptide is an independent risk factor for CVDs and its mortality (19). This effect is applied by increasing the vascular permeability to monocytes, their differentiation to macrophages, enhancing the phagocytosis of oxidized low-density lipoprotein (LDL), and converting to foam cells, which are pivotal factors in the formation of atherosclerotic lesions (44). Subsequently, C-peptide promotes the proliferation of smooth muscle cells and induces the release of pro-atherogenic factors such as cytokines, metalloproteinases, oxidants, and clotting agents such as the tissue plasminogen activator (tPA) (45, 46). In addition, the findings of past studies have shown that a higher serum level of C-peptide is associated with an increased risk of several types of malignancy, such as colorectal, pancreatic, and breast cancer (13, 47, 48).

### 4.4. Strengths and limitations

The present meta-analysis had some strengths. First, we have entered the number of significant studies that provided a relatively high sample size. Also, the countries where these studies were conducted were of good diversity. The assembly of these factors allowed us to obtain valid results from the composition of the data and to effectively discover the sources of heterogeneity between studies. Second, in CVD mortality, publication bias was not observed, but it was significant in all-cause mortality. In this context, two studies that were sources of bias were discovered, and after adjusting their effect, there was no significant change in the thousand ratios (4%). There are also some limitations in our meta-analysis. Some of the included studies have relatively small sample sizes. An unmeasured and residual confounding may lead to the inability to draw definitive conclusions. The included studies were adjusted for different covariates, and this meta-analysis pooled the adjusted HR that were adjusted for different variables. The analysis was also limited in terms of the quality of the single studies, and many other factors affecting CVD and all-cause mortality incidence could not be covered properly. Also, the included studies in our meta-analysis reported C-peptide as the quantitative and categorical variable that seems the combined obtained result is challengeable. For this reason, we conducted a subgroup analysis based on the type of exposure analysis (per 1 unit and highest vs. lowest category).

In conclusion, our study revealed that an increased level of serum C-peptide is positively associated with the risk of both CVD and all-cause mortality. Regarding the mentioned limitations, further large-scale studies and sufficient samples are needed to present a convincing link between C-peptide and the risk of CVD and all-cause mortality.

## Data Availability

The original contributions presented in the study are included in the article/**Supplementary Material**, further inquiries can be directed to the corresponding authors.
