# Supplementary material for: Serum C-peptide level and the risk of cardiovascular diseases mortality and all-cause mortality: a meta-analysis and systematic review
Source: Front Cardiovasc Med. 2023 Jul 7;10:1205481. doi: 10.3389/fcvm.2023.1205481 (PMC10360119; doi:10.3389/fcvm.2023.1205481)
Supplement: Supplementary file 1 [file Datasheet1.pdf]

LIST OF SUPPLEMENTARY MATERIALS

- **Table S1: The PICO criteria used for the present systematic review and meta-analysis.**
- **Table S2: Search Strategies used for different databases.**
- **Table S3: Quality assessment using New Castle - Ottawa Scale for cohort studies.**
- **Table S4: Sensitivity analyses after removing any study in the association between c-peptide with the risk of all-cause mortality.**
- **Table S5: Sensitivity analyses after removing any study in the association between c-peptide with the risk of cardiovascular disease mortality.**
- **Figure S1: Egger's funnel plot (with pseudo 95% confidence interval) depicting log RR (relative risk) against their corresponding standard error for assessing the presence of publication bias in studies that investigated the association between c-peptide with the risk of all-cause mortality(Fig A) and cardiovascular diseases mortality(Fig B).**
- **Figure S2: Meta-regression plot of hazard ratio and year of publication in studies that investigated the association between c-peptide with the risk of cardiovascular disease mortality.**
- **Figure S3: Preferred Reporting Items for Systematic Reviews and Meta-Analyses (PRISMA)**

Table S1. The PICO criteria used for the present systematic review and meta-analysis

PICO criteria	Description
**Patients**	Adult subjects
**Exposure**	Serum c-peptide level
**Comparison**	The highest category of c-peptide level versus the lowest category and per 1 SD or unit of c-peptide
**Outcome**	The risk of all-cause and CVD mortality

Table S2: Search Strategies used for different databases

databases	Search Strategy	Amount of articles
**PubMed**	(((cardiovascular*[tiab] OR heart diseas*[tiab] OR mortality*[tiab] OR death*[tiab] OR survival*[tiab] OR coronary artery*[tiab] OR overall mortality*[tiab]) OR ("Heart Diseases"[Mesh])) OR ("Mortality"[Mesh])) AND ("C-Peptide"[Mesh] OR proinsulin*[ti])	770
**Scopus**	((TITLE-ABS-KEY (mortality*)) OR (TITLE-ABS-KEY (survival*)) OR (TITLE-ABS-KEY (death*)) OR (TITLE-ABS-KEY (cardiovascular*)) OR (TITLE-ABS-KEY (heart diseas*)) OR (TITLE-ABS-KEY (coronary artery*)) OR (TITLE-ABS-KEY (overall mortality*))) AND ((TITLE-ABS-KEY ((c-peptid*))))	3403
**Web of science**	(TS=(overall mortality* OR death * OR mortality* OR survival* OR cardiovascular* OR coronary artery* OR heart diseas*)) AND TS=(c-peptide*)	2000

Table S3: Quality assessment using New Castle - Ottawa Scale for cohort studies[*]

	Selection				Comparability	Outcome			
**Study**	Representativeness of The exposed cohort	Selection of the non-Exposed cohort	Ascertainment of exposure	Demonstration that The outcome of Interest was not Present at start of The study	Comparability of Cohorts on the basis of design or analysis	Assessment of outcome	Was follow-up Long enough For the outcome to occur?	Adequacy of follow up of cohorts	Study score
Marx et al, 2013	*	*	*	*	**	*	*	*	9/9
Pikkemaat et al, 2019	*	*	*	*	**	*	*	*	8/9
Hirai et al, 2009	*	*	-	*	**	*	*	*	8/9
Patel et al, 2012	*	*	-	*	**	*	*	*	8/9
Min et al, 2013	*	*	*	*	**	*	*	-	8/9
Cardellini et al, 2017	*	*	*	*	**	*	*	-	8/9
Volkova et al, 2011	*	*	*	*	*	-	*	*	7/9
Bo et al, 2012	*	*	*	*	**	*	*	*	9/9
Griffith et al, 2011	*	*	*	*	**	*	-	*	8/9
Guercio et al, 2020	*	*	*	-	**	*	*	*	8/9
Irwin et al, 2011	*	*	*	*	**	*	*	-	8/9
Rovere et al, 2003	*	*	*	*	**	*	*	*	9/9
Schrauben et al, 2019	*	*	*	*	**	*	*	*	9/9
Wolpin et al, 2009	*	*	*	*	**	*	*	*	9/9
Zhu et al, 2020	*	*	*	*	**	*	-	*	8/9

** Study score less than 4 indicates low quality, a score of 4 to 6 represents moderate, and a score of more than 6 indicates as a high-quality*

Table S4: Sensitivity analyses after removing any study in the association between c-peptide with the risk of all-cause mortality.

**Study omitted**	**e^coef (95% confidence interval)**
Griffith	1.27 (1.13 – 1.41)
Pikkemaat	1.17 (1.09 – 1.27)
Patel	1.17 (1.08 – 1.27)
Min	1.18 (1.09 – 1.27)
Zhu	1.21 (1.12 – 1.32)
Schrauben	1.23 (1.12 – 1.35)
Irwin	1.20 (1.10 – 1.31)
Guercio	1.22 (1.12 – 1.33)
Marx	1.20 (1.10 – 1.30)
Bo	1.23 (1.12 – 1.34)
Hirai	1.23 (1.12 – 1.34)
Wolpin	1.20 (1.11 – 1.30)
Cardellini	1.23 (1.12 – 1.34)
Volkova	1.26 (1.14 – 1.40)
Combined	1.21 (1.11 – 1.32)

Table S5: Sensitivity analyses after removing any study in the association between c-peptide with the risk of cardiovascular diseases mortality.

Study omitted	e^coef (95% confidence interval)
Pikkemaat	1.30 (1.01 – 1.66)
Patel	1.35 (1.03 – 1.78)
Min	1.25 (1.01 – 1.54)
Marx	1.35 (1.03 – 1.79)
Bo	1.47 (1.12 – 1.93)
Rovere	1.46 (1.15 – 1.85)
Hirai	1.40 (1.02 – 1.94)
Hirai	1.43 (1.06 – 1.91)
Cardellini	1.38 (1.03 – 1.84)
Combined	1.38 (1.07 – 1.77)

Figure S1: Egger's funnel plot (with pseudo 95% confidence interval) depicting log RR (relative risk) against their corresponding standard error for assessing the presence of publication bias in studies that investigated the association between c-peptide with the risk of all-cause mortality(Fig A) and cardiovascular diseases mortality(Fig B).

Fig A:

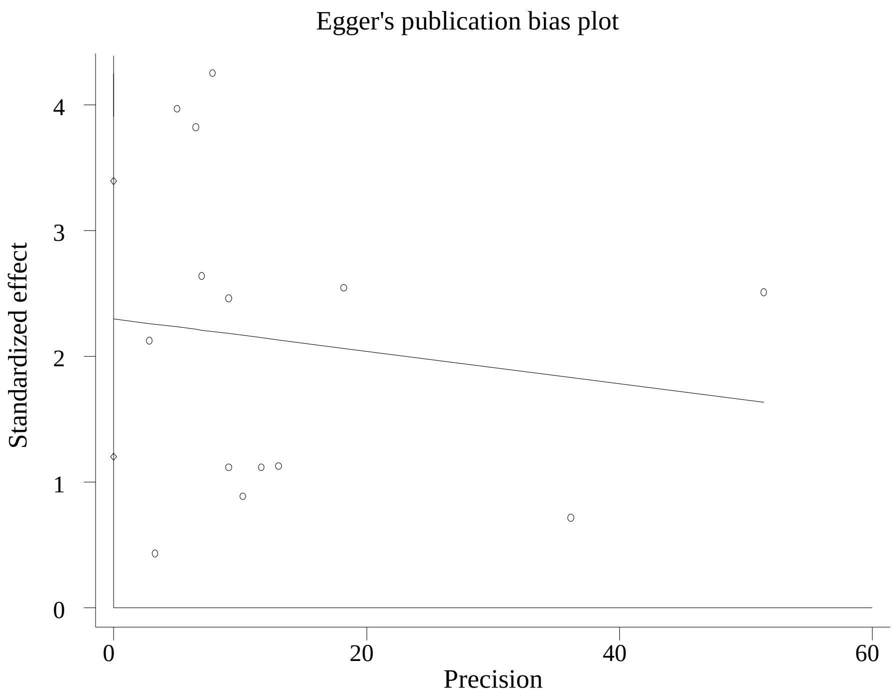

Fig B:

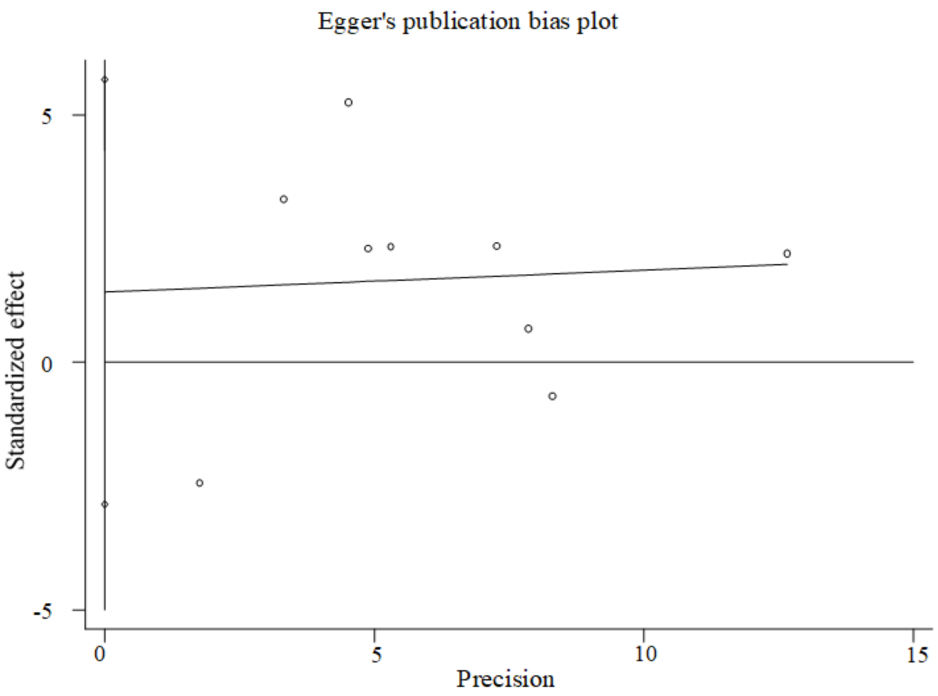

Figure S2: Meta-regression plot of hazard ratio and year of publication in studies that investigated the association between c-peptide with the risk of cardiovascular diseases mortality.

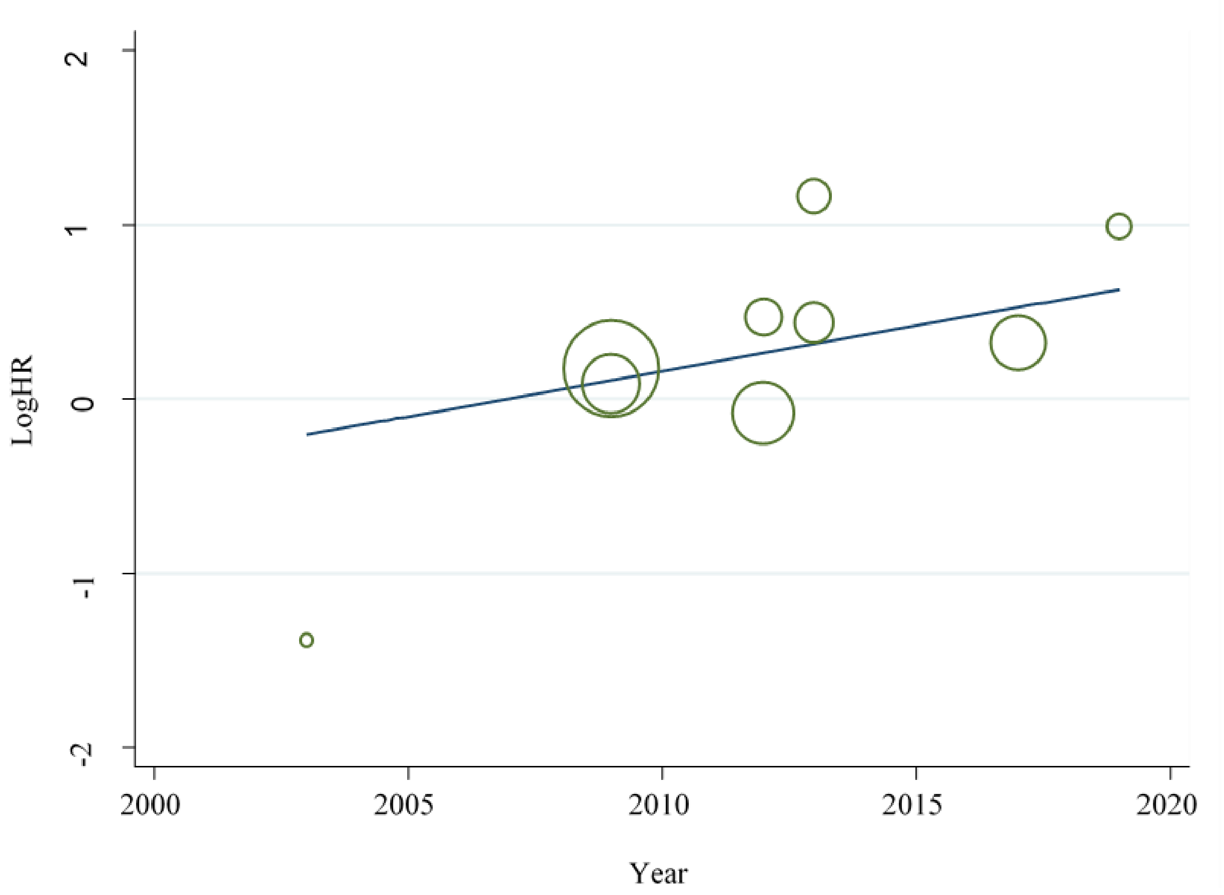

Figure S3: PRISMA checklist

Section/topic	#	Checklist item	Reported on page #
**TITLE**			
Title	1	Identify the report as a systematic review, meta-analysis, or both.	1
**ABSTRACT**			
Structured summary	2	Provide a structured summary including, as applicable: background; objectives; data sources; study eligibility criteria, participants, and interventions; study appraisal and synthesis methods; results; limitations; conclusions and implications of key findings; systematic review registration number.	2
**INTRODUCTION**			
Rationale	3	Describe the rationale for the review in the context of what is already known.	4
Objectives	4	Provide an explicit statement of questions being addressed with reference to participants, interventions, comparisons, outcomes, and study design (PICOS).	5
**METHODS**			
Protocol and registration	5	Indicate if a review protocol exists, if and where it can be accessed (e.g., Web address), and, if available, provide registration information including registration number.	5
Eligibility criteria	6	Specify study characteristics (e.g., PICOS, length of follow-up) and report characteristics (e.g., years considered, language, publication status) used as criteria for eligibility, giving rationale.	5,6
Information sources	7	Describe all information sources (e.g., databases with dates of coverage, contact with study authors to identify additional studies) in the search and date last searched.	5
Search	8	Present full electronic search strategy for at least one database, including any limits used, such that it could be repeated.	5
Study selection	9	State the process for selecting studies (i.e., screening, eligibility, included in systematic review, and, if applicable, included in the meta-analysis).	5,6
Data collection process	10	Describe method of data extraction from reports (e.g., piloted forms, independently, in duplicate) and any processes for obtaining and confirming data from investigators.	5,6
Data items	11	List and define all variables for which data were sought (e.g., PICOS, funding sources) and any assumptions and simplifications made.	5,6

Risk of bias in individual studies	12	Describe methods used for assessing risk of bias of individual studies (including specification of whether this was done at the study or outcome level), and how this information is to be used in any data synthesis.	6,7
Summary measures	13	State the principal summary measures (e.g., risk ratio, difference in means).	6
Synthesis of results	14	Describe the methods of handling data and combining results of studies, if done, including measures of consistency (e.g., I^2) for each meta-analysis.	6,7

Section/topic	#	Checklist item	Reported on page #
Risk of bias across studies	15	Specify any assessment of risk of bias that may affect the cumulative evidence (e.g., publication bias, selective reporting within studies).	6,7
Additional analyses	16	Describe methods of additional analyses (e.g., sensitivity or subgroup analyses, meta-regression), if done, indicating which were pre-specified.	6,7
**RESULTS**			
Study selection	17	Give numbers of studies screened, assessed for eligibility, and included in the review, with reasons for exclusions at each stage, ideally with a flow diagram.	7
Study characteristics	18	For each study, present characteristics for which data were extracted (e.g., study size, PICOS, follow-up period) and provide the citations.	Table1
Risk of bias within studies	19	Present data on risk of bias of each study and, if available, any outcome level assessment (see item 12).	Table2, 3
Results of individual studies	20	For all outcomes considered (benefits or harms), present, for each study: (a) simple summary data for each intervention group (b) effect estimates and confidence intervals, ideally with a forest plot.	Figure 2
Synthesis of results	21	Present results of each meta-analysis done, including confidence intervals and measures of consistency.	Figure 2
Risk of bias across studies	22	Present results of any assessment of risk of bias across studies (see Item 15).	10, 11 and Figure S1
Additional analysis	23	Give results of additional analyses, if done (e.g., sensitivity or subgroup analyses, meta-regression [see Item 16]).	9-11
**DISCUSSION**			
Summary of evidence	24	Summarize the main findings including the strength of evidence for each main outcome; consider their relevance to key groups (e.g., healthcare providers, users, and policy makers).	11

Limitations	25	Discuss limitations at study and outcome level (e.g., risk of bias), and at review-level (e.g., incomplete retrieval of identified research, reporting bias).	13, 14
Conclusions	26	Provide a general interpretation of the results in the context of other evidence, and implications for future research.	12, 13
**FUNDING**			
Funding	27	Describe sources of funding for the systematic review and other support (e.g., supply of data); role of funders for the systematic review.	15

From: Moher D, Liberati A, Tetzlaff J, Altman DG, The PRISMA Group (2009). Preferred Reporting Items for Systematic Reviews and Meta-Analyses: The PRISMA Statement. PLoS Med 6(7): e1000097. doi:10.1371/journal.pmed1000097

For more information, visit: **www.prisma-statement.org**.
