# Supplementary material for: Serum C-peptide level and the risk of cardiovascular diseases mortality and all-cause mortality: a meta-analysis and systematic review
Source: Front Cardiovasc Med. 2023 Jul 7;10:1205481. doi: 10.3389/fcvm.2023.1205481 (PMC10360119; doi:10.3389/fcvm.2023.1205481)
Supplement: Supplementary file 2 [file Table1.doc]

**Fig 1.** Flow diagram of selection of the published studies


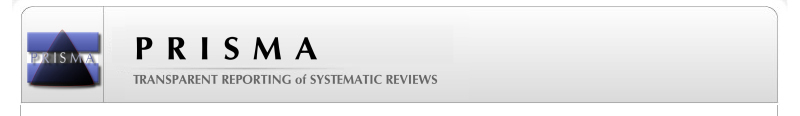
**PRISMA 2009 Flow Diagram**

**Screening**

**Included**

**Eligibility**

**Identification**

Records identified through database searching
(n =6173)

Records after duplicates removed
(n =4803)

Records screened
(n = 4803)

Records excluded
(n =4771)

Full-text articles assessed for eligibility
(n = 32)

Full-text articles excluded, with reasons
(n = 17)

Not relevant outcome(n=11)

Irrelevant (n=3)

Reported mean ± SD (n=2) and

Repetition (n=1)

Studies included in qualitative synthesis
(n =15)

Studies included in quantitative synthesis (meta-analysis)
(n = 15)
